# Nutrient deficiency patterns and all-cause and cardiovascular mortality in older adults with hypertension: a latent class analysis

**DOI:** 10.1186/s12889-024-19003-w

**Published:** 2024-06-10

**Authors:** YuJiao Sun, HuanRui Zhang, GuoXian Qi, Wen Tian

**Affiliations:** Department of Geriatric, The First Hospital of China Medical University, 155 Nanjing North Street, Heping Ward, Shenyang, 110001 NO China

**Keywords:** Nutrient deficiency, Mortality, Older adults, Hypertension, Latent class analysis (LCA)

## Abstract

**Background:**

Previous researches examining the impact of dietary nutrition on mortality risk have mainly focused on individual nutrients, however the interaction of these nutrients has not been considered. The purpose of this study was to identify of nutrient deficiencies patterns and analyze their potential impact on mortality risk in older adults with hypertension.

**Methods:**

We included participants from the National Health and Nutrition Examination Survey (NHANES) study. The latent class analysis (LCA) was applied to uncover specific malnutrition profiles within the sample. Risk of the end points across the phenogroups was compared using Kaplan–Meier analysis and Cox proportional hazard regression model. Multinomial logistic regression was used to determine the influencing factors of specific malnutrition profiles.

**Results:**

A total of 6924 participants aged 60 years or older with hypertension from NHANES 2003–2014 was followed until December 31, 2019 with a median follow-up of 8.7 years. Various nutrients included vitamin A, vitamin B1, vitamin B12, vitamin C, vitamin D, vitamin E, vitamin K, fiber, folate, calcium, magnesium, zinc, copper, iron, and selenium, and LCA revealed 4 classes of malnutrition. Regarding all-cause mortality, “Nutrient Deprived” group showed the strongest hazard ratio (1.42 from 1.19 to 1.70) compared with “Adequate Nutrient” group, followed by “Inadequate Nutrient” group (1.29 from 1.10 to 1.50), and “Low Fiber, Magnesium, and Vit E” group (1.17 from 1.02 to 1.35). For cardiovascular mortality, “Nutrient Deprived” group showed the strongest hazard ratio (1.61 from 1.19 to 2.16) compared with “Adequate Nutrient” group, followed by “Low Fiber, Magnesium, and Vit E” group (1.51 from 1.04 to 2.20), and “Inadequate Nutrient” group (1.37 from 1.03 to 1.83).

**Conclusions:**

The study revealed a significant association between nutrients deficiency patterns and the risk of all-cause and cardiovascular mortality in older adults with hypertension. The findings suggested that nutrients deficiency pattern may be an important risk factor for mortality in older adults with hypertension.

**Supplementary Information:**

The online version contains supplementary material available at 10.1186/s12889-024-19003-w.

## Introduction

With an increasing burden of global population aging, the prevalence of hypertension and hypertension-related mortality is significantly increasing, making it a critical public health concern [1–4]. Despite the increasing advancements in antihypertensive medication therapy, hypertension-related mortality still exceeded 10 million in 2019 [5]. It is of particular significance to enhance the prognosis and reduce the risk of premature death in older adults with hypertension. In addition to medication, adhering to a healthy dietary style is also an essential component of managing hypertension [6]. Epidemiological studies have indicated that certain types of food groups [7], dietary patterns [8], and diet quality [9] are associated with a reduced risk of mortality. The correlation between diet and mortality suggested that certain beneficial nutrients obtained through dietary intake might play a crucial role in reducing the risk of mortality [7, 10]. On the other hand, a deficiency in nutrients has been demonstrated to increase the risk of mortality [11].

Vitamins and minerals, as essential nutrients, play crucial roles in maintaining human health. The relationships between dietary vitamins and mortality have been established in prior studies, yet their findings remain controversial [12–16]. Dietary minerals, as indispensable components of foods, have been associated with many chronic diseases and mortality [17, 18], but no definitive conclusion has been reached. Dietary fiber is an important nutrient in a healthy diet, as recommended by nutritional guidelines [19]. We previously demonstrated that increased dietary fiber intake was associated with a decreased risk of mortality in older adults diagnosed with hypertension [20]. These researches on the relationship between dietary nutrition and mortality have primarily focused on individual nutrients. However, these dietary nutrients do not be taken isolated, the consideration of nutrient interactions is lacking due to the dietary diversity of humans. A recent study has indicated that there exist interactions between dietary iron and vitamins with mortality [21], which lends support to the notion of an interactive effect of nutrients on mortality.

It is of great importance to identify disparities in dietary nutrient profiles and their effects on the prognosis in older adults with hypertension, which will facilitate targeted interventions aimed at optimizing nutritional intake. Latent class analysis (LCA) is a person-centered statistical technique that utilizes data-driven method to identify relatively homogeneous sub-populations with similar characteristics. LCA has been described as a more logical and informative approach to investigate preference heterogeneity in the field of health [22]. LCA has been widely adopted in health and medical areas including behavioral sciences [23], physical diseases [24, 25] and psychology diseases [26]. This has contributed to the development of more rational policies for managing healthcare systems [22]. LCA should be an appropriate statistical method for evaluating the effect of dietary nutrients clustering on mortality in older adults with hypertension. However, to the best of our knowledge, no previous studies have been conducted to identify the nutrients deficiencies patterns (including vitamins, fiber, and minerals) in older adults with hypertension using LCA and their associations with mortality risk.

Through the utilization of the LCA method, this study aims to identify dietary nutrients deficiencies patterns in older adults with hypertension, based on National Health and Nutrition Examination Survey (NHANES) 2003–2014. Subsequently, the potential predictors of dietary nutrients deficiencies patterns will be analyzed to identify individuals who are at a higher risk of experiencing nutritional inadequacies. Lastly and most significantly, the confirmation of associations between dietary nutrient deficiencies patterns and mortality risk in older adults with hypertension is imperative.

## Methods

### Study design and patients

We included participants from NHANES study, which is a comprehensive nationwide survey of the U.S. non-institutionalized civilian for assessing their health and nutritional status. All protocols adhered to the approval of the National Center for Health Statistics Research Ethics Review Board, and participants provided written informed consent (source: https://www.cdc.gov/nchs/nhanes/irba98.htm). Data of older adult (≥ 60 years) with hypertension obtained from six NHANES cycles (2003–2004, 2005–2006, 2007–2008, 2009–2010, 2011–2012, and 2013–2014) was used, with a total sample size of 8,209 individuals. Following the exclusion of participants without 24-h dietary recall, follow-up outcome, and covariates, the final analytic sample encompassed 6,924 individuals. All the analyses conducted in this study strictly adhered to the analytic guidelines of NHANES.

### Nutritional assessment

Dietary data were acquired from the 'Dietary Interview-Total Nutrient Intakes' section of the NHANES study. This data was collected through two 24-h dietary recalls: an initial in-person dietary recall interview conducted at the Mobile Examination Center (MEC) and a subsequent interview conducted via telephone, typically occurring 3 to 10 days later. During these interviews, participants reported their food intake over the preceding 24-h period. In cases where participants completed both interviews, a mean daily intake value was calculated by averaging data from both interviews. Otherwise, the single dietary recall was used. To assess nutritional deficiencies, recommended daily intakes (Dietary Guidelines For Americans 2015–2020) were established for various nutrients, such as vitamin A, vitamin B1, vitamin B12, vitamin C, vitamin D, vitamin E, vitamin K, fiber, folate, calcium, magnesium, zinc, copper, iron, and selenium. Subsequently, participants were classified into two groups for each specific nutrient: (0) those who did not meet the minimum dietary intake and (1) those who met the minimum intake. These classifications were employed to identify latent profiles within the sample.

### All-cause and cardiovascular mortality

Our primary mortality outcomes encompassed all-cause and cardiovascular mortality. To achieve this, we established a connection between the NHANES database from 2003 to 2014 and the NHANES Public-use Linked Mortality Files, accessible at this source: https://www.cdc.gov/nchs/data-linkage/mortality-public.htm. Cardiovascular mortality was defined as any fatality attributed to cardiovascular disease, as identified by the ICD-10 codes (I00-09, 111, 113, and I20-51). The follow-up duration was calculated as the time elapsed from the date of the interview at MEC to the date of death, or it was subject to right-censoring at the end of the follow-up period on December 31, 2019.

### Covariates

Anthropometric and demographic characteristics, including age, gender, ethnicity (Non-Hispanic White, Non-Hispanic Black, Hispanic, and other race), education (less than high school, and above high school), marital status (married/with partner, and other), body mass index (BMI), and smoking status were analyzed, as well as comorbidity, such as hyperlipidemia, diabetes, cardiovascular disease.

### Statistical analysis

The LCA and other statistical analyses were conducted using Mplus (Version 8.3) and R software (Version 4.0.3). Considering the intricate multistage sampling design of NHANES, a sampling weight was constructed in accordance with NHANES guidelines. Statistical significance was determined at a threshold of *P* < 0.05.

1. The LCA was applied to identify a latent structure that categorizes a population into mutually exclusive and distinct homogeneous groups. For this study, we used LCA to uncover distinct malnutrition profiles within the sample. The analysis assessed participants' compliance with the minimum recommended daily intake levels of various nutrients, including vitamin A, vitamin B1, vitamin B12, vitamin C, vitamin D, vitamin E, vitamin K, fiber, folate, calcium, magnesium, zinc, copper, iron, and selenium (see Supplement File 1). To determine the number of classes, we compared model relied on various fit criteria. Models with low values for the Akaike Information Criterion (AIC), the Bayesian Information Criterion (BIC), and adjusted the Bayesian Information Criterion (aBIC) were deemed superior, signifying a better fit among competing models. The entropy value indicated how distinct the latent classes were in relation to one another, with values closer to one indicating clear classification. The Lo-Mendell-Rubin likelihood ratio test (LMR-LRT) was employed to assess the number of latent classes. If the probability value (P) was <0.05, the k model was considered superior. In the present analysis, a cluster number of 5 showed the model superior based on LMR-LRT (P<0.05), and a relative low AIC, BIC and aBIC value. However, 5-group clustering exhibits a relatively low frequency for smallest class, accounting for only 5.95%, and it was difficult to interpret the characteristics of each phenotype and makes it impractical for daily clinical application. Striking a balance between clinical interpretability and statistical soundness, we opted for a 4-group clustering in this study.
2. After we determined the optimal clusters, we designated the four latent class based on differences in nutrient deficiencies: 1) Adequate Nutrient; 2) Low Fiber, Magnesium, and Vit E; 3) Inadequate Nutrient; 4) Nutrient Deprived. Risk of the end points across the phenogroups was depicted by the weighted Kaplan-Meier curves and compared by the log-rank test. Taking the “Adequate Nutrient” group as the reference, we employed the weighted Cox proportional hazard regression models to evaluate the correlation between malnutrition profiles and all-cause and cardiovascular mortality. This analysis allowed us to estimate the hazard ratios (HRs) and 95% confidence intervals (CIs). We started with a crude model with no modifiable risk factors. We then introduced age, gender, and ethnicity as covariates in Model I, and further added marital status, education, BMI, smoking, hyperlipidemia, diabetes, cardiovascular in Model II. 
3. To determine whether the formation of malnutrition profiles depends on demographic information, anthropometric, and comorbidity, the weighted multinomial logistic regression was used. This method allows to evaluate specific associations between covariates and malnutrition profiles.

## Results

**Fig. 1:**
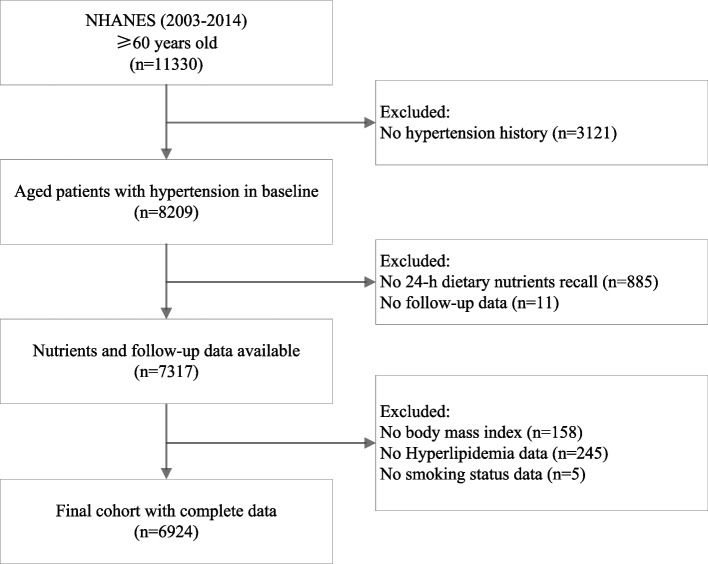
Flow chart of participants selection

**Fig. 2:**
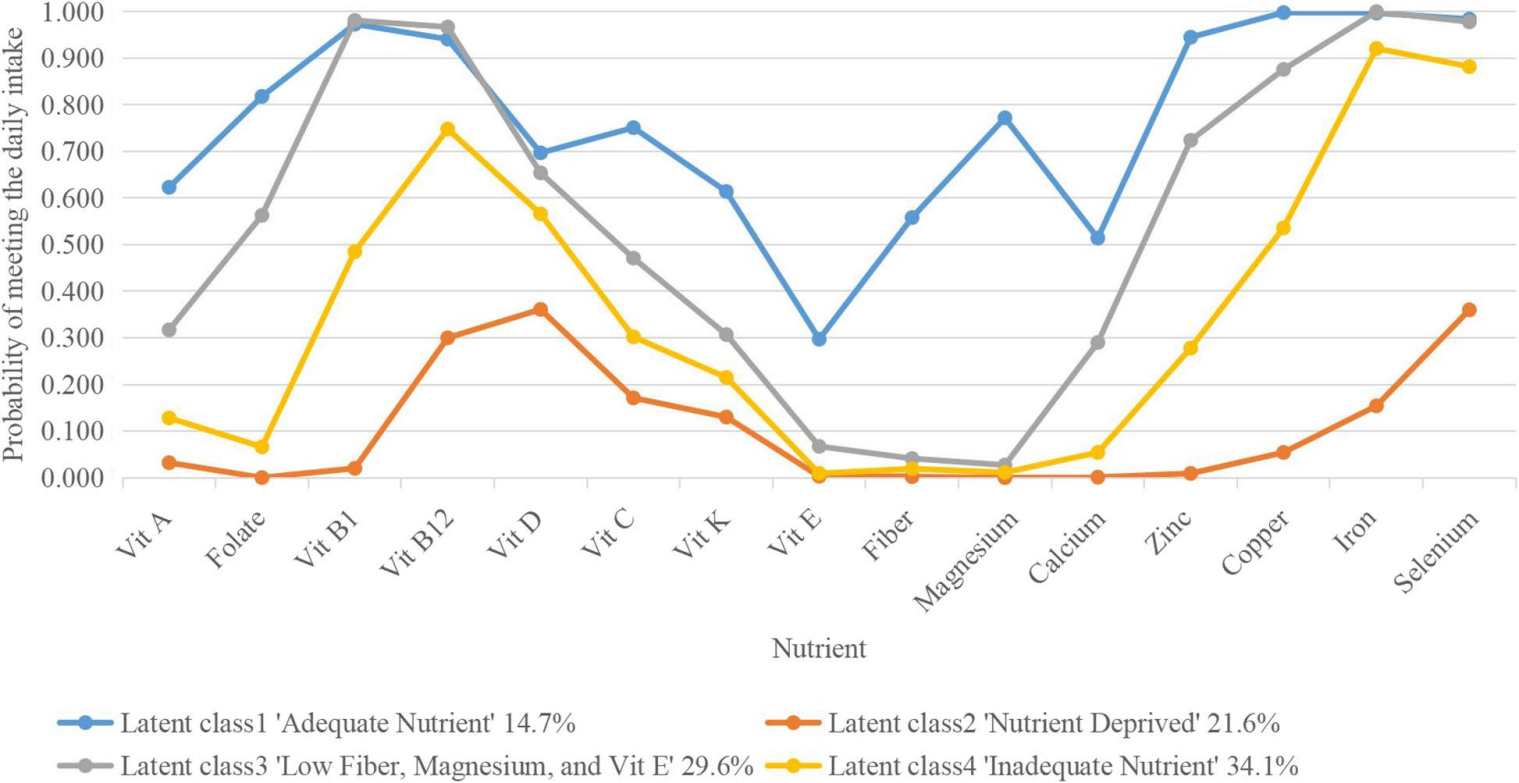
Pattern plot of 4-class profiles based on a latent class analysis

We utilized data from 6924 individuals aged 60 years or older with hypertension enrolled in the NHANES 2003–2014 (as shown in Fig. 1). For our LCA analysis, we focused on various nutrients, including vitamin A, vitamin B1, vitamin B12, vitamin C, vitamin D, vitamin E, vitamin K, fiber, folate, calcium, magnesium, zinc, copper, iron, and selenium, totaling 15 features. The fit statistics for LCA model are showed in Supplement File 2. In present analysis, striking a balance between clinical interpretability and statistical soundness, we ultimately chose for a 4-group clustering. Relative differences in various nutrient features among the four latent classes are depicted in Fig. 2, and corresponding prevalence of latent classes, and item-response probabilities of four latent classes are presented in Supplement File 3. The latent class 1 (*n* = 1019, 14.7% of the total sample) exhibited high comparative overall values in most nutrients respects and was designated as the “Adequate Nutrient” group. The latent class 2 (*n* = 1498, 21.6% of the total sample) represented individuals with the lowest likelihood of meeting recommended dietary intake, thus earning the label “Nutrient Deprived” group. The latent class 3 (*n* = 2049, 29.6% of the total sample) closely resembled the “Adequate Nutrient” group with the notable exception of lower levels of fiber, magnesium, and vitamin E, leading to its designation as the “Low Fiber, Magnesium, and Vit E” group. The latent class 4 (*n* = 2358, 34.1% of the total sample) was characterized by lower levels of fiber, magnesium, and vitamin E, but medium to high levels of other nutrients and it was designated as the “Inadequate Nutrient” group.

**Fig. 3:**
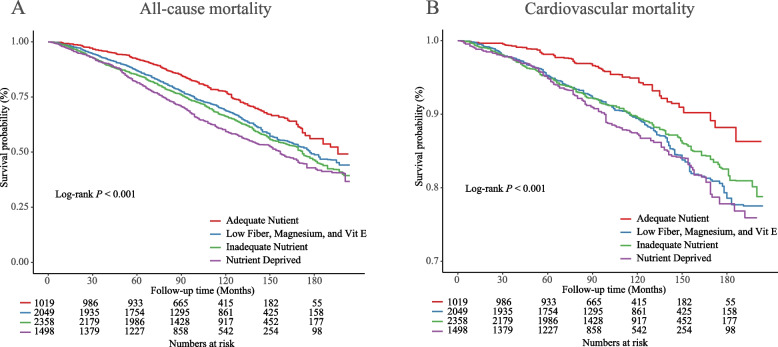
Kaplan–Meier curves were depicted to show all-cause and cardiovascular mortality by 4-class profiles

The weighted baseline characteristics of the participants are presented in Table 1. Among 6924 participants from US NHANES (weighted mean age 70.52 years, 56.3% female), 78.8% was Non-Hispanic White, 9.8% was Non-Hispanic Black, 2.8% was Mexican American/Hispanic, and 8.6% was other ethnicity. In present study, a total of 2742 deaths were recorded (766 death from cardiovascular disease) during a median follow-up period of 8.7 years. The weighted Kaplan–Meier curves were depicted based on malnutrition profiles at baseline (Fig. 3). Compared with “Adequate Nutrient” group, participants with poorer nutrients intake status tended to have a higher risk of all-cause and specific-cause mortality (Log-rank *P* < 0.001).

**Table 1:** The basic characteristics of hypertensive older adults in NHANES 2003–2014

Characteristics	Weighted Mean/Proportion	SE
Age, years^a^	70.52	0.14
Gender, female^b^	56.3	0.65
Ethnicity^b^		
Non-Hispanic White	78.8	1.27
Non-Hispanic Black	9.8	0.78
Mexican American/Hispanic	2.8	0.39
Other	8.6	0.73
Education, above high school^b^	75.9	1.03
Marital status, Married/with partner ^b^	60.4	0.91
Smoking, presence^b^	52.1	0.97
Body mass index, kg/m^2 a^	29.47	0.11
Hyperlipidemia, presence ^b^	55.6	0.74
Diabetes, presence^b^	27.1	0.69
Cardiovascular disease, presence^b^	27.8	0.88

^a^continuous variables were presented as weighted mean(SE); ^b^categorical variables were presented as weighted %(SE)

After adjusting for other covariates, including age, gender, ethnicity, marital status, education, BMI, smoking, hyperlipidemia, diabetes, and cardiovascular, the hazards ratios when older adult in “Low Fiber, Magnesium, and Vit E” group were compared with those in “Adequate Nutrient” group were 1.17 (1.02 to 1.35) for all-cause mortality, and 1.51 (1.04 to 2.20) for cardiovascular mortality; the hazards ratios of participants in “Inadequate Nutrient” group were 1.29 (1.10 to 1.50) for all-cause mortality, and 1.37 (1.03 to 1.83) for cardiovascular mortality; the hazards ratios of individuals in “Nutrient Deprived” group were 1.42 (1.19 to 1.70) for all-cause mortality, and 1.61 (1.19 to 2.16) for cardiovascular mortality (Table 2). Additionally, the hazard ratios in crude model and Model 1 were larger.

**Table 2:** The association of nutrient deficiency patterns with all-cause and cardiovascular mortality

	Crude model HR (95%CI), *P*-value	Model I HR (95%CI), *P*-value	Model II HR (95%CI), *P*-value
All-cause mortality			
Adequate Nutrient	ref	ref	ref
Low Fiber, Magnesium, and Vit E	1.38(1.18, 1.62), < 0.001	1.25(1.07, 1.45), 0.004	1.17(1.02, 1.35), 0.027
Inadequate Nutrient	1.51(1.29, 1.77), < 0.001	1.41(1.21, 1.63), < 0.001	1.29(1.10, 1.50), 0.001
Nutrient Deprived	1.80(1.48, 2.18), < 0.001	1.61(1.35, 1.93), < 0.001	1.42(1.19, 1.70), < 0.001
Cardiovascular mortality			
Adequate Nutrient	ref	ref	ref
Low Fiber, Magnesium, and Vit E	1.98(1.34, 2.93), 0.001	1.77(1.21, 2.59), 0.004	1.51(1.04, 2.20), 0.029
Inadequate Nutrient	1.83(1.33, 2.52), < 0.001	1.69(1.26, 2.26), < 0.001	1.37(1.03, 1.83), 0.028
Nutrient Deprived	2.25(1.61, 3.13), < 0.001	1.96(1.44, 2.67), < 0.001	1.61(1.19, 2.16), 0.002

Crude model adjusted for no variable

Model I adjusted for age, gender, and ethnicity

Model II adjusted for Model I plus marital status, education, BMI, smoking, hyperlipidemia, diabetes, cardiovascular disease

HR (95% CI), Hazard ratio and 95% confidence interval

Table 3 displays the factors associated with malnutrition profiles. These findings reveal distinct relationships between covariates and malnutrition profiles, particularly concerning class 1 (Adequate Nutrient). In contrast to the “Adequate Nutrient”, older adult in “Low Fiber, Magnesium, and Vit E” group were associated with being male, ethnicity, less education, BMI, and cardiovascular (all *P* < 0.05); older adult in “Inadequate Nutrient” group were associated with being older, ethnicity, less education, BMI, and cardiovascular (all *P* < 0.05); older adult in “Nutrient Deprived” group were associated with being older, female, ethnicity, less education, and cardiovascular (all *P* < 0.05).

**Table 3:** Correlates of nutrient deficiency patterns—multinomial logistic regression

Characteristics	Low Fiber, Magnesium, and Vit E vs. Adequate Nutrient	Inadequate Nutrient vs. Adequate Nutrient	Nutrient Deprived vs. Adequate Nutrient
	OR (95%CI), *P*-value		
Age	1.02(1.00, 1.03), 0.050	1.02(1.00, 1.03), 0.025	1.02(1.01, 1.04), 0.012
Sex			
male	ref	ref	ref
female	0.60(0.47, 0.75), < 0.001	0.84(0.67, 1.06), 0.148	1.51(1.18, 1.94), 0.001
Ethnicity			
Non-Hispanic White	ref	ref	ref
Non-Hispanic Black	1.20(0.89, 1.61), 0.226	2.15(1.57, 2.96), < 0.001	3.24(2.33, 4.50), < 0.001
Mexican American/Hispanic	1.05(0.67, 1.64), 0.834	1.38(0.90, 2.12), 0.142	2.31(1.50, 3.57), < 0.001
Other	0.58(0.42, 0.80), 0.001	0.97(0.71, 1.32), 0.845	1.52(1.11, 2.08), 0.009
Marital status			
Other	ref	ref	ref
Married/with partner	1.10(0.89, 1.37), 0.387	1.00(0.83, 1.22), 0.978	0.89(0.69, 1.15), 0.363
Education			
Less than high school	ref	ref	ref
Higher than high school	0.56(0.43, 0.72), < 0.001	0.50(0.39, 0.63), < 0.001	0.34(0.26, 0.45), < 0.001
Body mass index	1.03(1.02, 1.05), < 0.001	1.03(1.01, 1.05), 0.001	1.02(1.00, 1.04), 0.137
Smoking			
Absence	ref	ref	ref
Presence	0.94(0.77, 1.16), 0.580	0.85(0.68, 1.06), 0.149	0.98(0.77, 1.26), 0.887
Hyperlipidemia			
Absence	ref	ref	ref
Presence	1.08(0.89, 1.31), 0.440	1.02(0.84, 1.24), 0.827	1.03(0.82, 1.31), 0.775
Diabetes			
Absence	ref	ref	ref
Presence	1.24(0.98, 1.56), 0.074	1.17(0.93, 1.46), 0.180	1.28(1.00, 1.65), 0.052
Cardiovascular disease			
Absence	ref	ref	ref
Presence	1.37(1.09, 1.72), 0.007	1.62(1.30, 2.02), < 0.001	1.53(1.18, 1.98), 0.001

OR (95% CI), odds ratio and 95% confidence interval

## Discussion

The study investigated and established the dietary nutrient deficiency patterns in older adults with hypertension, identified potential predictors of dietary nutrients deficiencies, and analyzed the impact of these patterns on all-cause and cardiovascular mortality, based on NHANES 2003–2014. We identified four distinct patterns of dietary nutrient deficiencies in older adults with hypertension using the LCA method: Class 1—Adequate Nutrient, Class 2—Nutrient Deprived, Class 3—Low Fiber, Magnesium and Vitamin E, and Class 4—Inadequate Nutrient. Each pattern exhibits unique demographic and anthropogenic characteristics and varying mortality risks. The all-cause and cardiovascular mortality were found to be the lowest in class1 (Adequate Nutrient) and the highest in class 2 (Nutrient Deprived). In class 3 (Low Fiber, Magnesium, and Vitamin E), which closely resembled the “Adequate Nutrient” class but with lower levels of vitamin E, fiber, and magnesium, both all-cause and cardiovascular mortality were significantly higher than class 1 (Adequate Nutrient). The risk of all-cause and cardiovascular mortality significantly increased when the deficient of fiber, Magnesium, and Vitamin E, and gradually increased as the nutrients deficiencies increased. The study suggested the dietary nutrients deficiency patterns of vitamins, dietary fiber and minerals had a great effect on all-cause and cardiovascular mortality risk among older adults with hypertension, especially fiber, magnesium, and vitamin E.

To the best of our knowledge, this study represents the first establishment of nutritional deficiency patterns in a nationally representative sample of older adults with hypertension. The four classes exhibited disparities in meeting the minimum daily recommended intakes of vitamins, fiber, and minerals. In terms of meeting the minimum daily recommended nutrient intakes, the "Adequate Nutrient" class performed best, followed by the "Low Fiber, Magnesium, and Vit E" class. The "Inadequate Nutrient" and "Nutrient Deprived" classes had a higher proportion of individuals not reaching the minimum daily recommended intakes for most nutrients. The most notable disparity between the "Adequate Nutrient" and "Low Fiber, Magnesium, and Vit E" classes lies in the intake of fiber, magnesium, and vitamin E. The classes of "Nutrient Deprived," "Low Fiber, Magnesium, and Vit E," and "Inadequate Nutrient" exhibited a standard-achieving rate for fiber, magnesium, and vitamin E below 10%, indicating that these three nutrients are most likely to be deficient in older adults with hypertension. A recently published study has identified the patterns of nutritional deficiency and evaluated their effects on depression across all age groups in NHANES 2017–2018. The nutrients, including dietary fiber, folate, vitamin B1, vitamin B12, vitamin K, calcium, magnesium, iron, zinc, copper and selenium were utilized for the establishment of classes [27]. Similar to our study, the percentages of dietary fiber and magnesium that met the minimum daily recommended intake were less than 20%, whereas vitamin E was not included in their investigation [27]. Our nutrient profile contained a greater range of essential vitamins, including vitamins A, C, D and E in comparison to their study. This more accurately reflects the true status of nutrients and their interactions.

Analysis of predictors about nutrient deficiency classes revealed that age was a significant risk factor for three nutrient deficiency classes (the class 2, 3 and 4). Nutrition and diet survey showed the proportion of inadequate intake of essential nutrients increased with age [28]. The US nationally representative biochemical data, based on NHANES 2003–2006, showed that 30–36% of older adults suffer from one or more micronutrient deficiencies [29]. Low energy requirements, functional losses and socioeconomic factors devoted to inadequate nutrition in older adults, and the inadequate nutritional status would be further aggravated with advancing age [28, 30, 31]. Our study suggested that higher than high school was a significant protective factor of three nutrient deficiency classes. Education, as an important component of socioeconomic status, exhibited a positive correlation with nutritional status [27] and clinical prognosis [32]. In the study, it was observed that female exhibited a protective effect against class 3. Previous studies have indicated that females exhibit a greater concern for maintaining a healthy diet and nutrient intake, and consume more plant-based foods [33]. However, female is a risk factor for nutrient deprived. Our study focuses on the older adults with hypertension, and their age is the oldest in nutrient deprived class. We speculate that intake decreases significantly with age in older women, which may result in female as a risk factor in nutrient deprived class. It has been shown that overweight and obesity has adverse impact on the nutritional status of individuals. Overweight and obesity are considered as a malnutrition state, usually with important deficiencies in vitamins, minerals and dietary fiber [34]. Our result showed that BMI was identified as a risk factor for nutrients deficiencies classes, which was in line with previous studies. The study found a significant variation in the impact of ethnicity on nutrient deficiency classification. Consistent with previous findings, significant disparities in dietary patterns and quality have been established based on ethnicity and race [35–37]. The disparities in dietary components and nutrient intake might be attributed to variations in geography, socioeconomic status, environment, behavioral and lifestyle factors, as well as policy guidance among different ethnicities [38]. A major characteristic for cardiovascular disease is the significant losses of essential nutrients including vitamins, minerals, and dietary fiber [39–41]. Nutrient intervention is a pivotal component in the prevention and management of cardiovascular disease, with its significance becoming increasingly indispensable [42]. In our study, cardiovascular disease significantly increased the likelihood of belonging to the three classes of nutrient deficiency patterns. Overall, age, ethnicity, BMI, and cardiovascular disease may serve as potential predictors of nutrient deficiency; however, this association is reversed for females and those with higher education.

More significantly, the present study is the first to directly provide evidence on the association of nutrient deficiencies with all-cause and cardiovascular mortality in older adults with hypertension. It was obvious that older adults with hypertension who met most nutritional values in “Adequate Nutrient” class exhibited the lowest all-cause and cardiovascular-cause mortality rates, while those who failed to meet most nutritional values in the "Nutrient Deprived" class displayed a completely opposite trend in terms of all-cause and cardiovascular mortality. Our findings are consistent with previous research, which has demonstrated a significant association between the intake of dietary fiber, vitamins, and minerals and mortality rates. Conversely, inadequate intake of these nutrients is associated with an increased risk of mortality [16, 18, 20, 21, 43]. In the study, Class 3 "Low Fiber, Magnesium, and Vitamin E" ranks second in terms of nutrient sufficiency, following Class 1 "Adequate Nutrient." However, there exist significant disparities in all-cause and cardiovascular mortality between Class 1 and Class 3. It is noteworthy that participants in class 3 had greater opportunities to meet the minimal recommended intakes of nutrients, except for dietary fiber, magnesium, and vitamin E, compared to those in class 4. Adequate intake of these individual nutrients has been previously associated with reduced risks of all-cause and cause-specific mortality [18, 44]. However, the difference of the all-cause and cardiovascular mortality between class 3 and class 2, class 4 is minimal. More adequate intakes of these nutrients expect dietary fiber, magnesium, and vit E failed to significantly reduce the risk of all-cause and cardiovascular mortality in class 3, compared with class 2 and 4 in our study. However, the cardiovascular mortality is lower in class 4 than class 3, and a similar trend is also seen in cardiovascular mortality risk. Possibly it is due to the limited sample size, the cardiovascular deaths are much few, which caused deviation. The results suggested the intakes of fiber, magnesium, and vitamin E might play a critical role in all-cause and cardiovascular mortality risk among older adults with hypertension compared with other nutrients, when considering the holistic effects of diet nutrients on mortality.

Dietary fiber has been paid great attention to due to its distinct role in health recently. Dietary fiber has been confirmed to improve and delay many chronic diseases, and reduce the risk of mortality [19, 45]. The effects of dietary fiber on reducing mortality risk may be attributed to relieve the inflammation, improve overall metabolic health and develop healthy gut microflora [46]. Magnesium is involved in a variety of physiological functions, and is considered as a cofactor of hundreds of enzymes participated in in essential reactions in the body [47]. Humans get magnesium by consuming magnesium-rich foods to maintain magnesium homeostasis, but there is relatively common phenomenon of magnesium deficiency, 68% of US adult population have less than the recommended dietary allowance of magnesium [48]. Hypertension in old adults and magnesium deficit are two frequent coexisting conditions [49]. A meta-analysis based on 40 prospective cohort studies found the significantly protective effect of magnesium intake against stroke, heart failure, diabetes, and all-cause mortality, and a certain degree of dose-dependent, respectively [50]. Magnesium might reduce the mortality risk through exerting potential effects on antiplatelet, maintaining glucose and insulin homeostasis, improving lipid metabolism and endothelial function, enhancing vascular and myocardial contractility, keeping gene stability, and controlling inflammation [51]. Vitamin E is an important dietary antioxidant and anti-inflammatory vitamin, can inhibit LDL oxidation and prevent oxidative damage of the pathological process in many chronic diseases[16]. But the influence of vitamin E on the risk of many chronic diseases and mortality remains controversy, and there is no consistent conclusion. Forty-four studies were included in a meta-analysis of dietary vitamin E and risk of cardiovascular disease, stroke, cancer, and mortality, respectively [52]. The results of meta-analysis found dietary vitamin E was significantly associated with cardiovascular disease, stroke, cancer, and mortality in the nonlinear dose–response analysis [52]. In our study, the inadequate intake of dietary fiber, magnesium, and vitamin E significantly increased the all-cause and cardiovascular mortality risk in older adults with hypertension, the three nutrient combinations may play a positive role in its effect on risk of mortality. Dietary fiber, magnesium, and vitamin E should be considered as a marker of adherence to a healthy diet. Early nutritional intervention of increasing intake of dietary fiber, magnesium, and vitamin E might contribute to reduce the mortality risk in older adults with hypertension. But its exact mechanism remains to be studied further.

Our finding suggested the mortality risk of older adults with hypertension could not be attributed to individual nutrients, but the interactions of multiple nutrients. Most previous studies focused on a single nutrient might have overestimated or underestimated the actual impact of nutrients on mortality [12, 13, 53]. Recently, researchers have begun to look at the interaction of nutrients along with people's improved knowledge about nutrients. A study from NHANES 2003–2005 analyzed the effects of circulating vitamins’ co-exposure (vitamin A, D, E, C, B12 and B9) with all-cause, cardiovascular and cancer mortality risk, their results found the higher vitamin D was significantly associated with reduced mortality risk [54]. The study of dietary iron and vitamins (including vitamin A, B2, B6, C, E, and folic acid) in association with mortality found their interactions on mortality, the dietary intakes of iron can affect the relationship between vitamins and mortality [21]. These findings suggested we might should focus more on overall patterns of nutrients, which should be more significantly associated with mortality compared with individual nutrients. And the contribution of each of the nutrients to the mortality risk reduction might vary. Vitamins, dietary fiber and minerals, as essential nutrients for human beings, cannot be generated in vivo and must be achieved through daily diet, but until now, no data on the association of holistic intake of dietary vitamins, dietary fiber and minerals with mortality is available. Considering synergistic and cumulative effects of vitamins, dietary fiber and minerals, the present study analyzed the interaction of nutrients within overall deficiency profiles on mortality risk. Our results supported different mortality risk was found in different nutrients deficiency pattern.

There are also some limitations in the study. The data of nutrients intake was acquired from 24-h dietary recall interviews, which might cause self-reports desirability or memory bias. Moreover, extrapolating these findings to bloodstream nutrient levels could potentially lead to inaccurate conclusions and inappropriate dietary recommendations. Besides, the intake of nutrients was only obtained at baseline without assessing the impact of these changes on mortality risk. The studied population consisted of participants with hypertension aged 60 or over, we are not sure if the same conclusions apply to other age and disease groups. Our study based on these nutrient minimum daily intake recommendations, thus might be not prove that the relationship between these nutrients patterns and mortality risk when exceeding these nutrients minimum daily intake recommendations. Finally, some other potential excluded confounding might have influence on the results. Despite its limitations, this is the first study to classify dietary nutrients patterns and analyze these association with mortality risk as far as we know, based on a large nationally-representative sample of older adults with hypertension. Also, Using LCA to classify the dietary nutrients patterns can be considered the advantage of the study, LCA contributed to assess the interactions of various dietary nutrients and analyze them as a whole entity, instead of evaluating individual relationships among nutrients.

## Conclusion

The study identified the association of four nutrients deficiency patterns (including vitamins, fiber, and minerals) with all-cause and cardiovascular-cause mortality risk in older adults with hypertension; it provided important insights into how nutrients management strategies for older adults with hypertension might be targeted differently in nutrients deficiency patterns, instead of considering individual nutrients. Our results might contribute to a better understanding of the synergistic effect of various nutrients on mortality risk and provide evidence for supporting nutrients deficiency pattern screening and to develop the nutrients deficiency pattern-promoting management for older adults with hypertension. Older adults with hypertension might benefit from strategies targeting nutrients deficiency patterns management. Further studies are still needed to prove our results.

### Supplementary Information

Supplementary Material 1.

Supplementary Material 2.

Supplementary Material 3.

## Data Availability

Analytical data discussed in this manuscript are publicly accessible on the NHANES website [https://www.cdc.gov/nchs/nhanes/index.htm], and upon request, the analytical data will be provided, subject to application.

## Abbreviations

LCA: Latent class analysis
NHANES: National Health and Nutrition Examination Survey
MEC: Mobile Examination Center
BMI: Body mass index
AIC: Akaike Information Criterion
BIC: Bayesian Information Criterion
aBIC: Adjusted the Bayesian Information Criterion
LMR-LRT: Lo-Mendell-Rubin likelihood ratio test
HRs: Hazard ratios
CIs 95%: Confidence intervals
