# Supplementary material for: Nutrient deficiency patterns and all-cause and cardiovascular mortality in older adults with hypertension: a latent class analysis
Source: BMC Public Health. 2024 Jun 10;24:1551. doi: 10.1186/s12889-024-19003-w (PMC11163810; doi:10.1186/s12889-024-19003-w)
Supplement: Supplementary file 1 — Supplementary Material 1. [file 12889_2024_19003_MOESM1_ESM.docx]

| Supplement File 1 The sample meeting the Recommended Dietary Intake from 2015-2020. | | | | |
| --- | --- | --- | --- | --- |
| Nutrient | Recommended Dietary Intake | | Number | Percentage, % |
|  | Female | Male |  |  |
| Vit A, mg | 700 | 900 | 1640 | 23.7 |
| Folate, μg | 400 | 400 | 2138 | 30.9 |
| Vit B1, mg | 1.1 | 1.2 | 4167 | 60.2 |
| Vit B12, μg | 2.4 | 2.4 | 5155 | 74.5 |
| Vit D, μg | 1.5 | 1.5 | 3931 | 56.8 |
| Vit C, mg | 75 | 90 | 2707 | 39.1 |
| Vit K, μg | 90 | 120 | 1969 | 28.4 |
| Vit E, mg | 15 | 15 | 474 | 6.8 |
| Fiber, g | 22.4 | 28 | 723 | 10.4 |
| Magnesium, mg | 320 | 420 | 899 | 13 |
| Calcium, mg | 1200 | 1000 | 1248 | 18 |
| Zinc, mg | 8 | 11 | 3117 | 45 |
| Copper, μg | 0.9 | 0.9 | 4161 | 60.1 |
| Iron, mg | 8 | 8 | 5481 | 79.2 |
| Selenium, μg | 55 | 55 | 5634 | 81.4 |
