# Supplementary material for: Nutrient deficiency patterns and all-cause and cardiovascular mortality in older adults with hypertension: a latent class analysis
Source: BMC Public Health. 2024 Jun 10;24:1551. doi: 10.1186/s12889-024-19003-w (PMC11163810; doi:10.1186/s12889-024-19003-w)
Supplement: Supplementary file 2 — Supplementary Material 2. [file 12889_2024_19003_MOESM2_ESM.docx]

| Supplement File 2 Model selection. | | | | | | | | | |
| --- | --- | --- | --- | --- | --- | --- | --- | --- | --- |
| Model | loglikelihood | AIC | BIC | aBIC | Entropy | LMR-LRT | LMR-LRT-P | Absolute frequency for smallest class | Relative frequency for smallest class |
| 1 | -56443.576 | 112917.153 | 113019.794 | 112972.128 | - | - | - |  | - |
| 2 | -47717.15 | 95496.301 | 95708.426 | 95609.915 | 0.846 | 17452.852 | <0.001 | 3157 | 0.45595 |
| 3 | -45577.13 | 91248.261 | 91569.87 | 91420.515 | 0.824 | 4280.04 | <0.001 | 1724 | 0.24899 |
| **4** | **-45092.653** | **90311.307** | **90742.4** | **90542.201** | **0.777** | **968.954** | **<0.001** | **1019** | **0.14717** |
| 5 | -44852.329 | 89862.657 | 90403.234 | 90152.191 | 0.764 | 480.65 | 0.0003 | 412 | 0.0595 |
| 6 | -44691.352 | 89572.704 | 90222.765 | 89920.878 | 0.779 | 321.953 | 0.2639 | 381 | 0.05503 |
| AIC, the Akaike Information Criterion; BIC, the Bayesian Information Criterion; aBIC, adjusted the Bayesian Information Criterion; LMR-LRT, Lo-Mendell-Rubin likelihood ratio test. | | | | | | | | | |
