# Supplementary material for: Nutrient deficiency patterns and all-cause and cardiovascular mortality in older adults with hypertension: a latent class analysis
Source: BMC Public Health. 2024 Jun 10;24:1551. doi: 10.1186/s12889-024-19003-w (PMC11163810; doi:10.1186/s12889-024-19003-w)
Supplement: Supplementary file 3 — Supplementary Material 3. [file 12889_2024_19003_MOESM3_ESM.docx]

| Supplement File 3 Prevalence of latent classes, and item-response probabilities in models with four latent classes | | | | |
| --- | --- | --- | --- | --- |
| Item | Latent class1 'Adequate Nutrient' 14.7% | Latent class2 'Nutrient Deprived' 21.6% | Latent class3 'Low Fiber, Magnesium, and Vit E' 29.6% | Latent class4 'Inadequate Nutrient' 34.1% |
| Vit A | 0.623 | 0.032 | 0.317 | 0.128 |
| Folate | 0.818 | 0.000 | 0.563 | 0.066 |
| Vit B1 | 0.973 | 0.020 | 0.981 | 0.485 |
| Vit B12 | 0.941 | 0.300 | 0.967 | 0.748 |
| Vit D | 0.697 | 0.361 | 0.654 | 0.566 |
| Vit C | 0.751 | 0.171 | 0.471 | 0.302 |
| Vit K | 0.614 | 0.130 | 0.307 | 0.215 |
| Vit E | 0.297 | 0.003 | 0.067 | 0.009 |
| Fiber | 0.558 | 0.002 | 0.041 | 0.019 |
| Magnesium | 0.772 | 0.000 | 0.027 | 0.011 |
| Calcium | 0.514 | 0.001 | 0.290 | 0.054 |
| Zinc | 0.945 | 0.009 | 0.724 | 0.278 |
| Copper | 0.998 | 0.054 | 0.876 | 0.536 |
| Iron | 0.997 | 0.154 | 1.000 | 0.921 |
| Selenium | 0.984 | 0.360 | 0.978 | 0.882 |
| Item-response probabilities, the conditional probabilities of observing specific response patterns on the observed items within each latent class, indicating the likelihood of an individual in a particular latent class exhibiting a particular response pattern on each item. | | | | |
